# CRASH2 in Germany [ISRCTN86750102]

**DOI:** 10.1186/1745-6215-7-22

**Published:** 2006-06-21

**Authors:** CH Chung, A Freiberger, M Kalkum, SP Luntz, H Shakur, CM Seiler

**Affiliations:** 1 University of Heidelberg, Medical Faculty, Coordination Centre for Clinical Trials (KKS), Germany; 2 Kreiskrankenhaus Tirschenreuth, Department of Surgery, Germany; 3 University of London, London School of Hygiene and Tropical Medicine (LSHTM), UK; 4 University of Heidelberg, Medical Faculty, Study Centre of the German Surgical Society (SDGC), Germany

## 

In June 2005, the Study Centre of the German Surgical Society (SDGC) in Heidelberg, Germany [1] agreed to participate in the investigator initiated trial CRASH2 [2]. Regulatory and administrative affairs within Germany were assigned to the Coordination Centre for Clinical Trials (KKS) at the University of Heidelberg, Germany.

CRASH2 is a multinational trial sponsored by the London School of Hygiene and Tropical Medicine (LSHTM), Great Britain, which aims to determine the effect of the antifibrinolytic agent tranexamic acid on death and transfusion requirement in adult trauma patients with ongoing significant haemorrhage, or who are considered to be at risk of significant haemorrhage. In addition, the effect on the risk of non-fatal vascular events (either haemorrhagic or occlusive) will be assessed. So far, hospitals in more than 30 countries have been involved and over 1300 patients have been randomised.

The KKS and the SDGC recruited several collaborating hospitals and prepared the request for authorisation of CRASH2 to the competent authority in Germany, the BfArM (Bundesinstitut für Arzneimittel und Medizinprodukte) and the independent ethics committees. This process turned out to be complicated and did not match the requirements of other EU countries which had already approved the trial.

The main difference is the way in which German authorities and authorities of some other countries have interpreted the EU Directive (Directive 2001/20/EC) with regards to the need for 'adequate' indemnity for clinical trials [3]. As European legislation about what constitutes 'adequate' indemnity is not harmonised, somecompetent authorities as part of their assessment, will judge the adequacy of the insurance provided whereas others insist on a set limit.

The indemnity insurance for CRASH2 procured by the LSHTM for all participating hospitals throughout the world (except for the USA) did not comply with the limits required by the federal German drug law (AMG).

These set limits have resulted in the need to purchase different and separate limits for the states where prescribed limits apply and represent a disadvantage to a German participation.

In Germany, the required limit per person is 500,000 Euro and the limit per trial is up to 15,000,000 Euro. Some insurers do not want to expose themselves to this much of a risk for one trial. This has led to very few insurers willing to insure clinical trials in Germany and can make insurances for investigator initiated trials too expensive. This in turn can lead to the unethical situation where non-commercial clinical trials cannot take place.

For more than nine months the KKS and the SDGC have been trying to procure a separate insurance for CRASH2 in Germany. Unfortunately, these attempts have not been successful. The only German insurance that offered an indemnity insurance did not consider CRASH2 a mere pharmacological trial and therefore requested additional fees in order to insure surgical risks not associated with the treatment under investigation and finally offered a premium (cost: over 22,000 Euro) that would only cover the first 50 patients randomised in CRASH2. The fact that risks not associated with the trial intervention are assessed as part of the trial indemnity is not comprehensible to the KKS and to the SDGC. As a result, LSHTM is now trying to procure a separate indemnity insurance for Germany in Great Britain but as the insurance laws differ between the two countries, the terms and conditions obtained so far have not complied with article 40 of the AMG.

In dealing with insurers, it is the authors experience that some companies fear they would be legally held responsible to pay for all side effects which might occur during a trial treatment, regardless as to whether a side effect is caused by the study drug or not. If this is the position of the insurers, this will have a major impact on all clinical trials and this view should be open to public debate.

In Germany, several persons and institutions have been involved in CRASH2 so far. All participants are still eager to contribute to CRASH2. The participation of German patients in CRASH2 is very important since the results of this trial will be relevant to their future care.

In view of this, harmonisation of insurance requirements across the EU is urgently needed to alleviate these differences in order to promote the conduction of multinational clinical trials.

## Competing interests

The author(s) declare that they have no competing interests.

## References

[B1] http://www.springerlink.com/openurl.asp?genre=article&eissn=1435-2451&volume=390&issue=2&spage=171.

[B2] http://www.crash2.lshtm.ac.uk.

[B3] Goudsmit F, Rios C, Wadlund J (2005). Insuring International Clinical Trials: Navigating the Quirks and Avoiding the Quagmires. J BioLaw Bus.

